# Discrepancies in ICD-9/ICD-10-based codes used to identify three common diseases in cancer patients in real-world settings and their implications for disease classification in breast cancer patients and patients without cancer: a literature review and descriptive study

**DOI:** 10.3389/fonc.2023.1016389

**Published:** 2023-09-06

**Authors:** Nora Tu, Mackenzie Henderson, Meera Sundararajan, Maribel Salas

**Affiliations:** ^1^ Epidemiology, Clinical Safety and Pharmacovigilance, Daiichi Sankyo, Inc., Basking Ridge, NJ, United States; ^2^ Center for Real-World Effectiveness and Safety of Therapeutics (CREST), University of Pennsylvania Perelman School of Medicine, Philadelphia, PA, United States

**Keywords:** validation, international classification of diseases (ICD), comorbidities, breast cancer, methodology, Validation study

## Abstract

**Background:**

International Classification of Diseases, Ninth/Tenth revisions, clinical modification (ICD-9-CM, ICD-10-CM) are frequently used in the U.S. by health insurers and disease registries, and are often recorded in electronic medical records. Due to their widespread use, ICD-based codes are a valuable source of data for epidemiology studies, but there are challenges related to their accuracy and reliability. This study aims to 1) identify ICD-9/ICD-10-based codes reported in literature/web sources to identify three common diseases in elderly patients with cancer (anemia, hypertension, arthritis), 2) compare codes identified in the literature/web search to SEER-Medicare’s 27 CCW Chronic Conditions Algorithm (“gold-standard”) to determine their discordance, and 3) determine sensitivity of the literature/web search codes compared to the gold standard.

**Methods:**

A literature search was performed (Embase, Medline) to find sources reporting ICD codes for at least one disease of interest. Articles were screened in two levels (title/abstract; full text). Analysis was performed in SAS Version 9.4.

**Results:**

Of 106 references identified, 29 were included that reported 884 codes (155 anemia, 80 hypertension, 649 arthritis). Overall discordance between the gold standard and literature/web search code list was 32.9% (22.2% for ICD-9; 35.7% for ICD-10). The gold standard contained codes not found in literature/web sources, including codes for hypertensive retinopathy/encephalopathy, Page Kidney, spondylosis/spondylitis, juvenile arthritis, thalassemia, sickle cell disorder, autoimmune anemias, and erythroblastopenia. Among a cohort of non-cancer patients (N=684,376), the gold standard identified an additional 129 patients with anemia, 33,683 with arthritis, and 510 with hypertension compared to the literature/web search. Among a cohort of breast cancer patients (N=303,103), the gold standard identified an additional 59 patients with anemia, 10,993 with arthritis, and 163 with hypertension. Sensitivity of the literature/web search code list was 91.38-99.96% for non-cancer patients, and 93.01-99.96% for breast cancer patients.

**Conclusion:**

Discrepancies in codes used to identify three common diseases resulted in variable differences in disease classification. In all cases, the gold standard captured patients missed using the literature/web search codes. Researchers should use standardized, validated coding algorithms when available to increase consistency in research and reduce risk of misclassification, which can significantly alter the findings of a study.

## 1 Introduction

International Classification of Diseases (ICD) coding is one of the oldest efforts to systematically classify and track diseases and mortality (1). Its first edition (the International List of Causes of Death) was released in 1893, and there have since been many revisions to ICD coding led by the World Health Organization (2). The ICD Ninth Revision, Clinical Modification (ICD-9-CM) was adopted in the United States in 1979, and the Tenth Revision, Clinical Modification (ICD-10-CM) was adopted in the United States in 2015 (2). ICD-9-CM and ICD-10-CM coding are modified versions of the WHO’s ICD-9 and ICD-10 coding systems, and are used in a variety of healthcare settings in the United States. They are frequently used by health insurers for the reimbursement of claims related to health care services. They are also recorded in patients’ electronic medical records and are used by many disease registries to record disease state information (3, 4). The presence of ICD-9-CM and ICD-10-CM codes in such a variety of U.S. healthcare data sources has introduced an invaluable source of information for epidemiology studies (5, 6). In research settings, ICD-9-CM and ICD-10-CM codes have been used for many purposes, including classifying patients’ disease status, studying the natural history and outcomes of diseases, and documenting comorbidities (6, 7).

However, the use of ICD-9-CM and ICD-10-CM coding for research is also associated with challenges related to the accuracy and consistency of their use, largely due to widespread and variable usage of the codes in administrative claims in the United States. O’Malley et al. (2005) found several sources of error in their coding, including coder training and experience, quality-control processes in place at healthcare facilities, and intentional or unintentional coding errors (8). Similarly, Liebovitz and Fahrenbach (2018) suggested limitations due to physician time constraints, inability to find codes, and lack of coverage warnings leading physicians to choose different codes, among other limitations (9). Some studies have reported error rates in ICD-9-based coding up to 80% (8). Thus, researchers’ decisions regarding which codes to include in research can potentially have a large impact on study results.

There have been many approaches to address this issue. Some researchers have attempted to create and validate standardized coding algorithms that can be used to identify diseases accurately and reliably in a variety of databases. For example, in 2005, Quan and colleagues (10) created and evaluated several ICD-based coding algorithms to identify common comorbidities such as diabetes and chronic pulmonary disease. In the years since these results were published, many researchers have used these coding algorithms in their own research to accurately identify comorbid diseases (10). Alternatively, some organizations that create or maintain databases provide researchers with their own coding algorithms that researchers can use to identify diseases specifically in their database.

One example of this is the Surveillance, Epidemiology, and End Results (SEER)-Medicare database. SEER-Medicare is a linked database that includes claims data for patients enrolled in Medicare who have a cancer diagnosis. SEER-Medicare provides researchers with a code list (the 27 CCW Chronic Conditions Algorithm) that was developed within SEER-Medicare data and can be used to identify common comorbidities within these data (11). This code list includes not only ICD-9-CM and ICD-10-CM codes, but other codes as well, such as Healthcare Common Procedure Coding System (HCPCS) and Current Procedural Terminology (CPT) codes.

In this study, we utilized a SEER-Medicare breast cancer (BC) dataset to understand the implications of using different coding algorithms to identify common comorbidities in patients with BC. Using the 27 CCW Chronic Conditions algorithm provided by SEER-Medicare as the gold standard to identify comorbidities, we were able to evaluate the implications of using different, often simpler, algorithms that are commonly used for identification of comorbidities in research. For this study, we chose to focus on identification of three common comorbidities in elderly patients with BC: anemia, hypertension, and arthritis.

There were three primary objectives of this literature review and descriptive study. The first objective was to use published literature and online sources to identify and summarize ICD-based codes used to identify anemia, hypertension, and/or arthritis. The second objective was to systematically compare the ICD-based codes identified from the literature/web search to the ICD-9-CM and ICD-10-CM codes included in the SEER-Medicare 27 CCW Chronic Conditions Algorithm (gold standard) to evaluate their discordance. The third objective was to evaluate numerical differences in disease classification in cohorts of breast cancer and non-cancer SEER-Medicare patients using the literature/web search codes compared to the gold standard and determine sensitivity of the literature/web search code list.

## 2 Materials and methods

### 2.1 Study design

A literature search was performed in Embase (1980 – 22 February 2021) and Medline (1946 – 22 February 2021) to find literature that reported ICD-9/ICD-10-based codes used to identify at least one of three diseases of interest: anemia, hypertension, and/or arthritis (including both osteoarthritis, OA, and rheumatoid arthritis, RA). The search was limited to articles in English. The full literature search strategy is reported in **Supplementary Table 1**. Additional sources were evaluated for articles, including PubMed, references of articles retrieved in the literature search, the American Medical Association’s (AMA) official 2019 ICD-10-CM codebook (9), healthcare institution guidance publications (12, 13), and online ICD code look-up tools (14–16).

Publications were eligible for inclusion if they reported ICD-9/ICD-10-based codes used to identify at least one disease of interest, regardless of the primary objectives and methods of the publication. We did not limit inclusion of articles to ICD-9-CM and ICD-10-CM only; other modifications of ICD-coding were included as well. If a publication reported both ICD-based codes and other types of codes (e.g., HCPCS, CPT, or National Drug Codes), it was eligible for inclusion. However, only ICD-based codes were evaluated in this study and all other types of codes were excluded (due to feasibility concerns, inconsistencies in use, and limited usefulness in some databases).

Two levels of article screening were performed by one researcher. In level 1 screening, the titles and abstracts of identified publications were reviewed. Articles that were selected to move on after level 1 screening were then reviewed in level 2 screening, in which the full texts of the articles were reviewed. If there was uncertainty about the decision to include a specific publication, a second researcher was consulted.

The following data were extracted from all included articles: ICD-9/ICD-10-based codes for anemia, hypertension, and arthritis, and code descriptions when reported. If descriptions were not reported, they were extracted from ICD code look-up tools. One researcher performed the data extraction in Microsoft Excel and a second researcher performed quality control on the extracted data. Statistical analysis was performed in SAS Version 9.4.

### 2.2 Statistical methods

To address the first objective, we summarized the ICD-9/ICD-10-based codes identified from the literature/web search for each disease state and provided brief descriptions of these codes.

To address the second objective, we evaluated and summarized the extent to which the ICD-based codes identified in the literature/web search differed from the ICD-9-CM/ICD-10-CM codes in the SEER Medicare 27 CCW Chronic Conditions Algorithm. This was measured using *percent discordance. Concordant codes* were defined as ICD-based codes that were in both the 27 CCW Chronic Conditions Algorithm and the literature/web search code list. *Discordant codes* were defined as ICD-based codes found only in either the 27 CCW Chronic Conditions Algorithm or the literature/web search code lists, but not both. *Total codes* were defined as any codes found in either the 27 CCW Chronic Conditions Algorithm or the literature/web search (including both concordant and discordant codes). The *percent discordant* was defined as:


$$percent\;discordant=\;\frac{number\;of\;discordant\;codes}{total\;codes}\;x\;100\%$$

To address the third objective, we classified cohorts of non-cancer and BC SEER-Medicare patients (2008 – 2016) using the ICD-based codes found in the literature/web search and separately using the 27 CCW Chronic Conditions Algorithm to determine the difference in overall patient counts with each disease when using the different ICD-based code lists. For this analysis, one comprehensive literature/web search code list was created that included all ICD-based codes for any of the three diseases of interest found in any of the 29 references included herein from the literature review. The 27 CCW Chronic Conditions Algorithm was considered the gold standard for this study for multiple reasons, including that it was developed specifically for use in the dataset that we used for this study, and because the literature/web search code list was an aggregated list, and thus did not represent one specific list of codes and has not undergone any validation. Using the 27 CCW Chronic Conditions Algorithm as the gold standard, we calculated sensitivity for the literature/web search code lists for each of the three diseases.

## 3 Results

### 3.1 Literature search results

After all duplicates were removed, the literature search retrieved a total of 84 references. Twenty-two additional references were identified through other means, such as searching PubMed and reviewing references of articles identified in the literature search (12–33). Out of a total of 106 references identified, 29 references met the inclusion criteria and were included in this study (34–40). All ICD-9/ICD-10-based codes extracted from the included literature/web search are reported in **Tables 1A** and **B**. All tables report a lowercase *x* in a code to indicate a wildcard, meaning this digit can be replaced with any number. Unless otherwise noted, a code with *n* wildcard places after a base code includes all codes with up to *n* digits after the base code (e.g., M16.xx includes M16.x).

**Table 1A:** All ICD-9-based codes extracted from the literature/web search and SEER Medicare 27 CCW chronic conditions algorithm.

Disease	Reference	Literature/Web ICD-9-Based Codes	SEER-Medicare 27 CCW Chronic Conditions Algorithm ICD-9-CM Codes
Anemia	Elixhauser (32)	280.x, 281.x, 285.2x, 285.9, 648.2	280.x, 281.x, 282.xx, 283.xx, 284.xx, 285.xx
	Golinvaux (40)	280.1, 280.8, 280.9, 281.x, 285.2x	
	Nickel (17)	285.9	
	Other identified codes^1^ (14)	282.xx, 283.xx, 284.xx, 285.xx	
Hypertension	Elixhauser (32)	401.x, 402.xx, 403.xx, 404.xx, 405.xx, 642.0x, 642.1x, 642.2x, 642.7x, 642.9x	362.11, 401.x, 402.xx, 403.xx, 404.xx, 405.xx, 437.2
	Quan (33)	401.x, 402.xx, 403.xx, 404.xx, 405.xx	
	Lee (23)	401.x, 402.xx, 403.xx, 404.xx, 405.xx	
	Nickel (17)	401.x, 402.xx, 403.xx, 404.xx, 405.xx, 437.2, 642.0x, 642.1x, 642.2x, 642.7x, 642.9x	
	Vergara (35)	401.9	
Rheumatoid Arthritis	Kim (19)	714.xx	714.0, 714.1, 714.2, 714.3x, 715.xx, 720.0, 721.0, 721.1, 721.2, 721.3, 721.9x
	Lacaille (21)	714.xx	
	Widdifield (22)	714.xx	
	Chung (36)	714.xx	
	Huang (38)	714.xx	
	Bernatsky (29)	714.xx	
	BCBS (13)	714.0	
	Yang (30)	714.0	
	Maclean (18)	714, 714.0, 714.1, 714.2, 714.4, 714.8x	
	Hanly (20)	714.0, 714.1, 714.2	
Osteoarthritis	Gore (27)	715.xx	

^1^Other codes were identified through searching the AMA’s official codebook; Blue Cross Blue Shield (BCBS).

**Table 1B:** All ICD-10-based codes extracted from the literature/web search and SEER Medicare 27 CCW chronic conditions algorithm.

Disease	Reference	Literature/Web ICD-10-Based Codes	SEER-Medicare 27 CCW Chronic Conditions Algorithm ICD-10-CM Codes
Anemia	Elixhauser (32)	D51.x, D52.x, D53.x, D50.0, D50.8, D50.9	D50.x, D51.x, D52.x, D53.x, D55.x, D56.x, D57.00, D57.01, D57.02 D57.1, D57.20, D57.211, D57.212, D57.219, D57.3, D57.40, D57.411, D57.412, D57.419, D57.80, D57.811, D57.812, D57.819, D58.x, D59.x^2^, D60.x, D61.xxx, D62, D63.x, D64.xx
	Ghezala (34)	D51.x	
	Zalfani (39)	D50.0	
	Other identified codes^1^ (14–16)	D50.x, D55.x, D58.x, D59.xx, D61.xxx, D62, D63.x, D64.xx	
Hypertension	Elixhauser (32)	I10, I11.x, I12.x, I13.xx, I15.x,	I10, I11.x, I12.x, I13.xx, I15.x, I67.4, N26.2, H35.03x
	Quan (33)	I10, I11.x, I12.x, I13.xx, I15.x,	
	Optum (12)	I10.x, I11.x	
	Lee (23)	I10, I11.x, I12.x, I13.xx, I15.x,	
Rheumatoid Arthritis	Widdifield (22)	M05.xxx, M06.xxx	M05.0xx, M05.2xx, M05.3xx, M05.4xx, M05.5xx, M05.6xx, M05.7xx^3^, M05.8xx^4^, M05.9, M06.xxx^5^, M08.xxx^6^
	Huang (38)	M05.xxx, M06.xxx	
	Bernatsky (29)	M05.xxx	
	BCBS (13)	M05.4xx, M05.5xx, M05.7xx, M05.8xx, M05.9, M06.0xx, M06.2xx, M06.3xx, M06.8xx, M06.9	
	Hanly (20)	M05.xxx, M06.0xx, M06.8xx, M06.9	
	Luque Ramos (25)	M05.xxx, M06.xxx	
	Curtis (37)	M05.xxx, M06.xxx	
	Fautrel (28)	M05.xxx, M06.xxx	
Osteoarthritis	French (31)	M15.x, M16.xx, M17.xx, M18.xx, M19.xxx	M15.x, M16.xx, M17.xx, M18.xx, M19.xxx^7^, M45.x, M47.xxx^8^, M48.8Xx^9^
	Barnabe (26)	M15.x, M16.xx, M17.xx, M18.xx, M19.xxx	
	Postler (24)	M16.x, M17.xx	
	Other identified codes^1^ (14)	M15.x, M16.xx, M17.xx, M18.xx, M19.xxx	

^1^Other codes were identified through searching the AMA official codebook and/or online code look-up tools; ^2^This includes D59.1, a nonbillable code; ^3^excluding M05.7A; ^4^excluding M05.8A; ^5^excluding M06.0A, M06.4, M06.8A; ^6^excluding M08.0A, M08.2A, M08.4A, M08.9A; ^7^excluding M19.19; ^8^excluding M47.14, M47.15, M47.16; ^9^Capital X indicates that the X is part of the code syntax, whereas a lowercase x indicates a wildcard.

### 3.2 Discordant code findings

#### 3.2.1 Overall discordance

Overall, 884 total codes were identified from either the literature/web search or SEER Medicare 27 CCW Chronic Conditions Algorithm: 180 were ICD-9-based and 704 were ICD-10-based codes. Of the total codes, 155 (17.5%) were for anemia, 80 (9.1%) were for hypertension, and 649 (73.4%) were for arthritis. There were 291 discordant codes found between the literature/web search code lists and 27 CCW Chronic Conditions Algorithm: 40 discordant ICD-9-based codes and 251 discordant ICD-10-based codes. This resulted in an overall discordance of 32.9% (22.2% for ICD-9-based codes and 35.7% for ICD-10-based codes) between the literature/web codes and the 27 CCW Chronic Conditions Algorithm. Discordant code findings are reported in **Tables 2A** and **B**.

**Table 2A:** Discordant ICD-9-based codes with code descriptions.

	ICD-9-Based Code	Brief Code Descriptions
Only in literature/web search		
*Anemia*	648.2	Anemia complicating pregnancy, childbirth or the puerperium
*Hypertension*	642.0x, 642.1x, 642.2x, 642.7x, 632.9x	Certain codes for hypertension complicating pregnancy, childbirth or the puerperium
*Arthritis (RA/OA)*	714, 714.4, 714.8, 714.81, 714.89, 714.9	Chronic postrheumatic arthropathy; other specified inflammatory polyarthropathies; unspecified inflammatory polyarthropathy
Only in SEER-Medicare 27 CCW Chronic Conditions Algorithm		
*Anemia*	N/A	
*Hypertension*	362.11	Hypertensive retinopathy
*Arthritis (RA/OA)*	720.0, 721.0, 721.1, 721.2, 721.3, 721.9x	Ankylosing spondylitis; certain spondylosis and allied disorders; spondylosis of unspecified site

**Table 2B:** Discordant ICD-10-based codes with code descriptions.

	ICD-10-Based Codes	Brief Code Descriptions
Only in literature/web search		
Anemia	D59.10, D59.11, D59.12, D59.13, D59.19	Other autoimmune hemolytic anemias
Hypertension	N/A	
Arthritis	M05.1xx^1^, M05.8A, M06.0A, M06.4	Rheumatoid lung disease with RA; other RA with RF of other specified site; RA without RF of other specified site; inflammatory polyarthropathy
	M19.09, M19.19, M19.29	Primary OA of other specified site; post-traumatic OA of other specified site; secondary OA of other specified site
Only in SEER-Medicare 27 CCW Chronic Conditions Algorithm		
Anemia	D56.x, D57.00, D57.01, D57.02, D57.1, D57.20, D57.211, D57.212, D57.219, D57.3, D57.40, D57.411, D57.412, D57.419, D57.80, D57.811,812,819, D59.1, D60.x	Thalassemia; certain sickle cell disorders; other autoimmune hemolytic anemia^2^; Acquired pure red cell aplasia (erythroblastopenia)
Hypertension	H35.03x, I67.4, N26.2	Hypertensive retinopathy; hypertensive encephalopathy; Page kidney
Arthritis	M08.0xx^3^, M08.1, M08.2xx^4^, M08.3, M08.4xx^5^, M08.8xx, M08.9xx^6^, M45.x, M47.0xx, M47.10, M47.11, M47.12, M47.13, M47.2x, M47.8xx, M47.9, M48.8Xx^7^	Certain juvenile RAs; juvenile ankylosing spondylitis; juvenile rheumatoid polyarthritis (seronegative); certain pauciarticular juvenile RA; other/unspecified juvenile arthritis; ankylosing spondylitis; other spondylosis with radiculopathy; other/unspecified spondylosis

^1^Excluding M05.19; ^2^not a billable code; ^3^excluding M08.0A; ^4^excluding M08.2A; ^5^excluding M08.4A; ^6^excluding M08.9A; ^7^Capital X indicates that the X is part of the code syntax, whereas a lowercase x indicates a wildcard.

#### 3.2.2 Anemia discordance

A total of 59 ICD-9-based anemia codes were identified from either the literature/web search or SEER Medicare 27 CCW Chronic Conditions Algorithm. Of these, there was one discordant code that was found in the literature/web search but not the 27 CCW Chronic Conditions Algorithm (**Table 2A**). This resulted in an overall discordance of 1.7% for ICD-9-based anemia codes. A total of 96 ICD-10-based anemia codes were identified from either the literature/web search or 27 CCW Chronic Conditions Algorithm. Of these, there were 35 discordant codes (30 of which were found only in the 27 CCW Chronic Conditions Algorithm and 5 of which were found only in the literature/web search; **Table 2B**). This resulted in an overall discordance of 36.5% for ICD-10-based anemia codes.

#### 3.2.3 Hypertension discordance

A total of 60 ICD-9-based hypertension codes were identified from either the literature/web search or the SEER Medicare 27 CCW Chronic Conditions Algorithm. Of these, there were 26 discordant codes (1 of which was found only in the 27 CCW Chronic Conditions Algorithm and 25 of which were only found in the literature/web search; **Table 2A**). This resulted in an overall discordance of 43.3% for ICD-9-based hypertension codes. A total of 20 ICD-10-based hypertension codes were identified from either the literature/web search or the 27 CCW Chronic Conditions Algorithm. Of these, there were 6 discordant codes (all of which were only found in the 27 CCW Chronic Conditions Algorithm; **Table 2B**). This resulted in an overall discordance of 30% for ICD-10-based hypertension codes.

#### 3.2.4 Arthritis discordance

For the arthritis code analysis, RA and OA were grouped together to be consistent with the SEER Medicare 27 CCW Chronic Conditions Algorithm, which includes only one overall group for arthritis. A total of 61 ICD-9-based arthritis codes were identified from either the literature/web search or the 27 CCW Chronic Conditions Algorithm. Of these, there were 13 discordant codes (7 were found only in the 27 CCW Chronic Conditions Algorithm and 6 were found only in the literature/web search; **Table 2A**). This resulted in an overall discordance of 21.3% for ICD-9-based arthritis codes. A total of 588 ICD-10-based arthritis codes were identified from either the literature/web search or the 27 CCW Chronic Conditions Algorithm. Of these, there were 210 discordant codes (182 were found only in the 27 CCW Chronic Conditions Algorithm and 28 were found only in the literature/web search; **Table 2B**). This resulted in an overall discordance of 35.7% for ICD-10-based arthritis codes.

### 3.3 Most frequently identified codes

The most frequent concordant ICD-9/ICD-10-based codes overall (i.e., those identified in both the literature/web search and the SEER-Medicare 27 CCW Chronic Conditions Algorithm), are reported in **Supplementary Table 2**. The most frequently identified anemia codes were for unspecified anemia, anemia of chronic illness or blood loss, and deficiency anemias (including iron and vitamin B12). The most frequently identified hypertension codes were for malignant or benign essential/primary hypertension, hypertensive heart disease, hypertensive chronic kidney disease (CKD), hypertensive heart disease and CKD, and secondary hypertension. The most frequently identified arthritis codes were for rheumatoid arthritis and variations thereof (e.g., with visceral involvement, with rheumatoid myopathy; **Supplementary Table 2**), osteoarthritis and variations thereof (e.g., of the hip, of the knee; **Supplementary Table 2**), rheumatoid bursitis or nodules, Felty’s syndrome, and adult-onset Still’s Disease.

The most frequently identified discordant codes found only in literature/web sources are listed in **Supplementary Table 3**. The most commonly found discordant ICD-9-based codes in the literature/web search included certain hypertensive disorders associated with pregnancy and childbirth and certain arthropathies/polyarthropathies (**Supplementary Table 3**). The most common discordant ICD-10-based codes in the literature/web search included certain codes for rheumatoid lung disease with RA, RA of unspecified sites, inflammatory polyarthropathy, and certain codes for OA of unspecified sites.

### 3.4 Classification of non-cancer and breast cancer patient cohorts in the SEER-Medicare database

Finally, to address the third objective of this study we evaluated the numerical differences in disease classification in two cohorts of patients in SEER-Medicare (non-cancer patients and BC patients) using the literature/web search codes compared to the SEER-Medicare 27 CCW Chronic Conditions Algorithm codes. These results are presented in **Tables 3A**, **B**. For non-cancer patients, the 27 CCW Chronic Conditions Algorithm identified 129 additional patients with anemia (p=0.83), 510 additional patients with hypertension (p=0.27), and 33,683 additional patients with arthritis (p<0.0001) that were not identified using the literature/web code list. Using the 27 CCW Chronic Conditions Algorithm as the gold standard, the comprehensive literature/web search code list had a 99.96% sensitivity to identify anemia in non-cancer patients, 99.91% sensitivity to identify hypertension in non-cancer patients, and 91.38% sensitivity to identify arthritis (including both OA and RA) in non-cancer patients. For BC patients, the 27 CCW Chronic Conditions Algorithm identified 59 additional patients with anemia (p=0.88), 163 additional patients with hypertension (p=0.66), and 10,993 additional patients with arthritis (p<0.0001) that were not identified using the literature/web code list. Using the 27 CCW Chronic Conditions Algorithm as the gold standard, the comprehensive literature/web search code list had a 99.96% sensitivity to identify anemia in BC patients, 99.92% sensitivity to identify hypertension in BC patients, and 93.01% sensitivity to identify arthritis in BC patients.

**Table 3A:** Total number of non-cancer SEER-Medicare patients (N=684,376) classified with each disease of interest using codes found in the literature/web search code list compared to the SEER-Medicare 27 CCW chronic conditions algorithm.

Disease	ICD Code Version	Number of non-cancer patients identified		Absolute difference	Sensitivity^2^	p-value^3^
		Literature/ web search	27 CCW Chronic Conditions Algorithm ^1^			
**Anemia**	ICD-9	300,876 (44.0%)	300,876 (44.0%)	0 (0)	–	1.00
	ICD-10	112,643 (16.5%)	113,128 (16.5%)	485 (0.1%)	–	0.26
	Any	330,644 (48.3%)	330,773 (48.3%)	129 (<0.1%)	99.96%	0.83
**Hypertension**	ICD-9	515,380 (75.3%)	515,845 (75.4%)	465 (0.1%)	–	0.36
	ICD-10	310,697 (45.4%)	311,195 (45.5%)	498 (0.1%)	–	0.39
	Any	548,324 (80.1%)	548,834 (80.2%)	510 (0.1%)	99.91%	0.27
**Arthritis**	ICD-9	324,569 (47.4%)	356,329 (52.1%)	31,760 (4.6%)	–	<0.0001
	ICD-10	143,426 (21.0%)	164,825 (24.1%)	21,399 (3.1%)	–	<0.0001
	Any	357,280 (52.2%)	390,963 (57.1%)	33,683 (4.9%)	91.38%	<0.0001

^1^Considered the gold standard for this study; ^2^Sensitivity refers to the sensitivity of the literature/web search code list when compared to the gold standard, the SEER-Medicare 27 CCW Chronic Conditions Algorithm; ^3^P-value based on Chi-square test

**Table 3B:** Total number of SEER-Medicare breast cancer patients N=303,103 classified with each disease of interest using codes found in the literature/web search code list compared to the SEER-Medicare 27 CCW chronic conditions algorithm.

Disease	ICD Code Version	Number of breast cancer patients identified		Absolute difference	Sensitivity^2^	p-value^3^
		Literature/ web search	27 CCW Chronic Conditions Algorithm ^1^			
**Anemia**	ICD-9	120,792 (39.9%)	120,792 (39.9%)	0 (0)	–	1.00
	ICD-10	52,584 (17.4%)	52,789 (17.4%)	205 (0.1%)	–	0.49
	Any	134,900 (44.5%)	134,959 (44.5%)	59 (0.0%)	99.96%	0.88
**Hypertension**	ICD-9	188,462 (62.2%)	188,610 (62.2%)	148 (0.1%)	–	0.70
	ICD-10	125,619 (41.4%)	125,800 (41.5%)	181 (0.1%)	–	0.64
	Any	203,026 (67.0%)	203,189 (67.0%)	163 (0.1%)	99.92%	0.66
**Arthritis**	ICD-9	131,529 (43.4%)	141,881 (46.8%)	10,352 (3.4%)	–	<0.0001
	ICD-10	66,675 (22.0%)	74,775 (24.7%)	8,100 (2.7%)	–	<0.0001
	Any	146,263 (48.3%)	157,256 (51.9%)	10,993 (3.6%)	93.01%	<0.0001

^1^Considered the gold standard for this study; ^2^Sensitivity refers to the sensitivity of the literature/web search code list when compared to the gold standard, the SEER-Medicare 27 CCW Chronic Conditions Algorithm; ^3^P-value based on Chi-square test

## 4 Discussion

A total of 884 codes were identified for anemia, hypertension, and arthritis. The majority of these codes were ICD-10-based codes (n=704), and the remainder were ICD-9-based codes (n=180). The discrepancy between number of codes in the ninth and tenth revisions was expected, given that there are almost five times as many ICD-10-CM codes as there are ICD-9-CM codes, largely due to differences in grouping and specificity between the ICD versions (6). The most common codes identified for anemia were for anemias of chronic illness or blood loss, unspecified anemias, and deficiency anemias. The most common codes identified for hypertension were for malignant or benign essential/primary hypertension, secondary hypertension, and hypertensive heart disease and/or hypertensive CKD. Finally, the most common codes for arthritis were for OA and variations thereof, RA and variations thereof, rheumatoid bursitis or nodules, Felty’s syndrome, and adult-onset Still’s Disease.

When the literature/web search code lists were compared to the SEER-Medicare 27 CCW Chronic Conditions Algorithm, there was variable discordance. Discordance for all codes was less than 50% (overall discordance was 32.9%), and higher discordance was observed for hypertension compared to either anemia or arthritis. Discordance for ICD-9-based codes ranged from 1.7% - 43.3% and discordance for ICD-10-based codes ranged from 30% - 36.5%. There were several codes included in the 27 CCW Chronic Conditions Algorithm that were not found in literature/web sources. These included certain codes for hypertensive retinopathy/encephalopathy, Page kidney, thalassemia, sickle cell disorders, autoimmune hemolytic anemia, erythroblastopenia, spondylitis/spondylosis, and juvenile arthritis conditions. On the other hand, the most common codes found only in the literature/web search included certain codes related to hypertensive disorders of pregnancy/childbirth, certain arthropathies/polyarthropathies, rheumatoid lung disease with RA, and RA of unspecified sites, (**Supplementary Table 3**).

There are many possible reasons for the differences between the codes included in the literature/web search code list and the 27 CCW Chronic Conditions Algorithm. Specific codes included in any given study may be driven largely by the population of interest. This is demonstrated clearly by the fact that pregnancy-related hypertensive disorders were not found in the 27 CCW Chronic Conditions Algorithm. Because the SEER-Medicare database primarily contains information about older adults (≥65 years old), codes related to pregnancy are less relevant in these patients, which may be why they were excluded. Interestingly, when examining codes found only in the 27 CCW Chronic Conditions Algorithm, there were several codes related to juvenile arthritis. As previously noted, SEER-Medicare includes data on mostly older individuals, so the rationale for including these codes in the code list is unclear. It is possible that since some types of juvenile arthritis are chronic diseases that persist into adulthood, they may remain relevant in older populations (41).

Furthermore, the exact codes used in a study may be based on the specific database being used, or based on previous research that has validated the use of specific codes to identify the disease of interest. As an example, a 2011 article by Kim et al. (19) performed a validation of several code lists to identify RA in Medicare claims data. Since this initial validation, this paper has been cited by over 150 articles, many of which used one of Kim et al.’s code lists to identify RA in their own research (19, 42–45). This indicates that a researcher’s decision about which codes to use may be based on previous work done to validate those codes in the same or similar databases.

A third potential reason for the differences seen may be due to variable consultation of clinical or coding experts when developing codes lists for specific diseases. When examining the codes found in the literature/web search and the SEER-Medicare 27 CCW Chronic Conditions Algorithm, each contained codes that did not explicitly match the disease name, but may have been included because a clinical expert deemed them appropriate. For example, under the scope of arthritis, the 27 CCW Chronic Conditions Algorithm includes codes for spondylosis and adult-onset Still’s disease. Professionals in medical coding and clinicians who specialize in a particular area of practice may be knowledgeable about common coding practices and diseases that share common features and may be able to use this knowledge to ensure face validity of code lists (46, 47).

Finally, another possible reason for the differences observed may be due to variation in the use of specific codes over time. Common ICD-9-/ICD-10-based coding practices or reimbursement policies for any given disease state may change over time, and this would in turn necessitate a change in the codes used to identify the given disease in a healthcare database. In addition, the code version used in the United States changed in 2015 from ICD-9-CM to ICD-10-CM. Thus, depending on the years included in a specific study, it may be necessary to include one or both of these code versions. These issues may also account for some of the differences in code lists observed in this study.

Regardless of the specific reasons for the variation in coding algorithms, the differences can result in important differences in patient classification. When we classified two cohorts of patients in SEER-Medicare, the literature/web search code list had between 91.38% – 99.96% sensitivity in identifying non-cancer patients and 93.01% - 99.96% sensitivity in identifying BC patients with the three diseases of interest. While the overall sensitivity was high, it should be noted that the sensitivity for the code lists used in individual studies may have been significantly lower than the overall sensitivity, given that we combined all 29 literature/web search code lists into one list for analysis. Interestingly, percent discordance did not necessarily correspond to lower sensitivity. While the highest discordance was identified for hypertension, the lowest overall sensitivity was seen for arthritis: the SEER-Medicare 27 CCW Chronic Conditions Algorithm identified a significant additional number of patients with arthritis in the non-cancer cohort (33,683 additional patients; p<0.0001) and in the BC cohort (10,993 additional patients p<0.0001) that were not identified with the literature/web search codes.

Using the 27 CCW Chronic Conditions Algorithm as the gold standard, the literature/web search code list misclassified a significant number of patients with arthritis. Because these codes are often used to assign patients to exposure or outcome groups, or used for subgroup analyses in epidemiology studies, issues of misclassification can affect the clinical interpretation of a study’s results. Whether this misclassification is differential or non-differential may depend on the study design and data source used. If misclassification occurs proportionally between the groups being compared to each other, this will result in non-differential misclassification and will bias the study results towards the null. The extent to which this is an issue for any particular study will depend largely on the disease of interest, its common coding practices, and the database used. However, the differences can be substantial, and this example offers a clear illustration of why researchers must carefully evaluate and determine which codes to include in their research.

This study has a few limitations. The gold standard (the 27 CCW Chronic Conditions Algorithm) and the ICD-10-CM coding system are frequently updated. We used the version of the 27 CCW Chronic Conditions Algorithm that was developed using data through 2016, aligning with the specific dataset used in this study. For this reason, we were able to use it as a gold standard for this study, but this algorithm has since been updated and our results may not reflect the most recent algorithms or ICD-10-CM coding. This study focused on the evaluation of how the literature/web search code list performed against the gold standard, but we were unable to evaluate the performance of the gold standard itself. It should also be noted that this algorithm undergoes continual updating to reflect the most current coding practices and understanding of the relevant disease states. In addition, definitions used to determine disease status in epidemiology studies are not limited to codes (e.g., ICD-9-CM or ICD-10-CM codes), but may incorporate other rules. These can include requirements for patients to have more than one code recorded for the disease, potentially at prespecified time intervals. Though not directly evaluated in the current study, articles that were included in our literature review varied extensively in their definitions of arthritis. Kim et al. (19) required patients to have at least two or three diagnosis codes for RA. Lacaille et al. (21) required at least two physician visits more than two months apart with a diagnosis code for RA. In contrast, French et al. (31) required only one diagnosis code for OA. Finally, Postler et al. (24) required an outpatient diagnosis of OA in at least two quarters of a single calendar year. These differences in coding algorithms must also be considered when determining the appropriate way to identify and classify patients’ disease status.

## 5 Conclusions

Although it may not be feasible to develop one coding algorithm to identify a specific disease for use across all databases, there is considerable room for improvement in the development of valid coding algorithms and increased consistency of their use in research. Researchers should carefully evaluate what codes to include in their research, and consider the potential implications of these decisions. If significant misclassification occurs because invalid coding algorithms are used to identify patients, this may bias the results of a study and call into question their clinical utility. It is advisable that researchers provide justification for their inclusion and exclusion of certain codes in their publications. Finally, if validated coding algorithms or validated ICD-9/ICD-10-based codes are available for use, researchers should use them in their research. Future work is needed to develop and validate coding algorithms for use in specific databases.

## Data availability statement

The data analyzed in this study is subject to the following licenses/restrictions: SEER-Medicare data are not public use data files. Requests to access these datasets should be directed to SEER-Medicare, SEERMedicare@imsweb.com.

## Ethics statement

The studies involving humans were approved by SEER-Medicare Review Committee. The studies were conducted in accordance with the local legislation and institutional requirements. Written informed consent for participation was not required from the participants or the participants’ legal guardians/next of kin in accordance with the national legislation and institutional requirements.

## Author contributions

NT contributed to the development of the research project, contributed to drafting of study materials including statistical analysis plan and study protocol, contributed to statistical analysis of data and quality control, critically reviewed drafts and approved the final manuscript. MH contributed to the development of the research project, contributed to drafting of all study materials, provided input into the statistical analysis plan, contributed to drafting of manuscript and critical review, approved final manuscript. MeS contributed to drafting of study materials, conducted statistical analysis of the data and quality control, contributed to the drafting of the manuscript, and critically reviewed drafts and approved final manuscript. MaS contributed to the development of the research project, provided input into the statistical analysis plan and interpretation of results, critically reviewed drafts and approved the final manuscript. All authors contributed to the article and approved the submitted version.

## References

[1] International Statistical Classification of Diseases and Related Health Problems (ICD). Geneva, Switzerland: World Health Organization (2021). Available at: https://www.who.int/standards/classifications/classification-of-diseases.

[2] ICD-9-CM Code Set. Salt Lake City, Utah, USA: AAPC. Available at: https://www.aapc.com/resources/medical-coding/icd9.aspx#:~:text=The%20current%20version%20used%20in%20the%20United%20States,%20input%20of%20providers,%20payers,%20and%20other%20key%20stakeholders.

[3] Thought Leadership Team Editorial Staff / AAPC ICD-10 and CMS eHealth: What’s the Connection? Centers for Medicare & Medicaid Services (2013). Available at: https://www.cms.gov/Medicare/Coding/ICD10/Downloads/ICD-10andCMSeHealth-WhatstheConnection_071813remediated[1].pdf.

[4] ICD-O-3 Coding Materials. National Cancer Institute. Available at: https://seer.cancer.gov/icd-o-3/.

[5] Classifications. World Health Organization. Available at: https://www.who.int/standards/classifications.

[6] The Role of the ICD-10 in Epidemiology. Louisville, KY, USA: Radius Anesthesia of Kentucky PLLC (2020). Available at: https://radiusky.com/icd-10-epidemiology/.

[7] International Classification of Diseases, (ICD-10-CM/PCS) Transition - Background. Centers for Disease Control and Prevention (2015). Available at: https://www.cdc.gov/nchs/icd/icd10cm_pcs_background.htm.

[8] O'Malley KJ Cook KF Price MD Wildes KR Hurdle JF Ashton CM . Measuring diagnoses: ICD code accuracy. Health Serv Res (2005) 40(5 Pt 2):1620–39. doi: 10.1111/j.1475-6773.2005.00444.x PMC1361216 16178999

[9] Liebovitz DM Fahrenbach J . COUNTERPOINT: is ICD-10 diagnosis coding important in the era of big data? No. Chest (2018) 153(5):1095–8. doi: 10.1016/j.chest.2018.01.034 29476710

[10] Quan H Sundararajan V Halfon P Fong A Burnand B Luthi JC . Coding algorithms for defining comorbidities in ICD-9-CM and ICD-10 administrative data. Med Care (2005) 43(11):1130–9. doi: 10.1097/01.mlr.0000182534.19832.83 16224307

[11] Chronic Conditions Data Warehouse. Baltimore, Maryland, USA: Centers for Medicare and Medicaid Services (2022).

[12] Hypertension coding tool. Optum (2019). Available at: https://myuha.org/wp-content/uploads/2020/05/Optum_Insider_Hypertension_Coding_Tool-11-20-19.pdf.

[13] Rheumatology ICD-10-CM Coding Tip Sheet: Overview of Key Chapter Updates for Rheumatology. Blue Cross Blue Shield of Michigan. Available at: https://www.bcbsm.com/content/dam/public/Providers/Documents/help/faqs/icd10-tipsheet-rheumatology.pdf.

[14] ICD-10-CM 2019: The Complete Official Codebook. 1 ed. Chicago, Illinois: American Medical Association (2018). Available at: https://books.google.com/books/about/ICD_10_CM_2019_the_Complete_Official_Cod.html?id=A0V0tgEACAAJ

[15] ICD10Data.com. Available at: https://www.icd10data.com.

[16] ICD9Data.com. Available at: http://www.icd9data.com/.

[17] Nickel KB Wallace AE Warren DK Ball KE Mines D Fraser VJ . Modification of claims-based measures improves identification of comorbidities in non-elderly women undergoing mastectomy for breast cancer: a retrospective cohort study. BMC Health Serv Res (2016) 16(a):388. doi: 10.1186/s12913-016-1636-7 27527888 PMC4986377

[18] MacLean CH Louie R Leake B McCaffrey DF Paulus HE Brook RH . Quality of care for patients with rheumatoid arthritis. JAMA (2000) 284(8):984–92. doi: 10.1001/jama.284.8.984 10944644

[19] Kim SY Servi A Polinski JM Mogun H Weinblatt ME Katz JN . Validation of rheumatoid arthritis diagnoses in health care utilization data. Arthritis Res Ther (2011) 13(1):R32. doi: 10.1186/ar3260 21345216 PMC3241376

[20] Hanly JG Thompson K Skedgel C . The use of administrative health care databases to identify patients with rheumatoid arthritis. Open Access Rheumatol Res Rev (2015) 7:69–75. doi: 10.2147/OARRR.S92630 PMC5045118 27790047

[21] Lacaille D Anis AH Guh DP Esdaile JM . Gaps in care for rheumatoid arthritis: a population study. Arthritis Rheumatol (2005) 53(2):241–8. doi: 10.1002/art.21077 15818655

[22] Widdifield J Bombardier C Bernatsky S Paterson JM Green D Young J . An administrative data validation study of the accuracy of algorithms for identifying rheumatoid arthritis: the influence of the reference standard on algorithm performance. BMC Musculoskelet Disord (2014) 15:216. doi: 10.1186/1471-2474-15-216 24956925 PMC4078363

[23] Lee DC Feldman JM Osorio M Koziatek CA Nguyen MV Nagappan A . Improving the geographical precision of rural chronic disease surveillance by using emergency claims data: a cross-sectional comparison of survey versus claims data in Sullivan County, New York. BMJ Open (2019) 9(11):e033373. doi: 10.1136/bmjopen-2019-033373 PMC6887089 31740475

[24] Postler A Ramos AL Goronzy J Günther KP Lange T Schmitt J . Prevalence and treatment of hip and knee osteoarthritis in people aged 60 years or older in Germany: an analysis based on health insurance claims data. Clin Interv Aging (2018) 13:2339–49. doi: 10.2147/CIA.S174741 PMC6241868 30532524

[25] Luque Ramos A Redeker I Hoffmann F Callhoff J Zink A Albrecht K . Comorbidities in patients with rheumatoid arthritis and their association with patient-reported outcomes: results of claims data linked to questionnaire survey. J Rheumatol (2019) 46(6):564–71. doi: 10.3899/jrheum.180668 30647170

[26] Barnabe C Hemmelgarn B Jones CA Peschken CA Voaklander D Joseph L . Imbalance of prevalence and specialty care for osteoarthritis for first nations people in Alberta, Canada. J Rheumatol (2015) 42(2):323–8. doi: 10.3899/jrheum.140551 25433531

[27] Gore M Tai KS Sadosky A Leslie D Stacey BR . Clinical comorbidities, treatment patterns, and direct medical costs of patients with osteoarthritis in usual care: a retrospective claims database analysis. J Med Econ (2011) 14(4):497–507. doi: 10.3111/13696998.2011.594347 21682606

[28] Fautrel B Cukierman G Joubert JM Laurendeau C Gourmelen J Fagnani F . Healthcare service utilisation costs attributable to rheumatoid arthritis in France: Analysis of a representative national claims database. Joint Bone Spine (2016) 83(1):53–6. doi: 10.1016/j.jbspin.2015.02.023 26671705

[29] Bernatsky S Dekis A Hudson M Pineau CA Boire G Fortin PR . Rheumatoid arthritis prevalence in Quebec. BMC Res Notes (2014) 7:937. doi: 10.1186/1756-0500-7-937 25527187 PMC4417509

[30] Yang DH Huang JY Chiou JY Wei JC . Analysis of socioeconomic status in the patients with rheumatoid arthritis. Int J Environ Res Public Health (2018) 15(6):1194. doi: 10.3390/ijerph15061194 29875338 PMC6024906

[31] French ZP Torres RV Whitney DG . Elevated prevalence of osteoarthritis among adults with cerebral palsy. J Rehabil Med (2019) 51(8):575–81. doi: 10.2340/16501977-2582 31282980

[32] Elixhauser A Steiner C Harris DR Coffey RM . Comorbidity measures for use with administrative data. Med Care (1998) 36(1):8–27. doi: 10.1097/00005650-199801000-00004 9431328

[33] Quan H Khan N Hemmelgarn BR Tu K Chen G Campbell N . Validation of a case definition to define hypertension using administrative data. Hypertension (2009) 54(6):1423–8. doi: 10.1161/HYPERTENSIONAHA.109.139279 19858407

[34] Ben Ghezala I Arendt JF Erichsen R Zalfani J Gammelager H Frøslev T . Positive predictive value of the diagnosis coding for vitamin B12 deficiency anemia in the Danish National Patient Register. Clin Epidemiol (2012) 4:333–8. doi: 10.2147/CLEP.S38229 PMC3526860 23271924

[35] Vergara VA . Identification of ICD-9 codes associated with scleroderma renal crisis. Clin Exp Rheumatol (2014) 32(2):S115.

[36] Chung CP Rohan P Krishnaswami S McPheeters ML . A systematic review of validated methods for identifying patients with rheumatoid arthritis using administrative or claims data. Vaccine (2013) 31(Suppl 10):K41–61. doi: 10.1016/j.vaccine.2013.03.075 24331074

[37] Curtis JR Xie F Zhou H Salchert D Yun H . Use of ICD-10 diagnosis codes to identify seropositive and seronegative rheumatoid arthritis when lab results are not available. Arthritis Res Ther (2020) 22(1):242. doi: 10.1186/s13075-020-02310-z 33059732 PMC7560310

[38] Huang S Huang J Cai T Dahal KP Cagan A Stratton J . Impact of international classification of diseases 10th revision codes and updated medical information on an existing rheumatoid arthritis phenotype algorithm using electronic medical data. Arthritis Rheumatol (2018) 70(Supplement 10). Available at: https://acrabstracts.org/abstract/impact-of-international-classification-of-diseases-10th-revision-codes-and-updated-medical-information-on-an-existing-rheumatoid-arthritis-phenotype-algorithm-using-electronic-medical-data/#:~:text=We%20observed%20that%20an%20existing%20RA%20algorithm%20trained,including%20ICD10%20had%20a%20minimal%20impact%20on%20classification.

[39] Zalfani J Frøslev T Olsen M Ben Ghezala I Gammelager H Arendt JF . Positive predictive value of the International Classification of Diseases, 10th edition diagnosis codes for anemia caused by bleeding in the Danish National Registry of Patients. Clin Epidemiol (2012) 4:327–31. doi: 10.2147/CLEP.S37188 PMC3516453 23236255

[40] Golinvaux NS Bohl DD Basques BA Grauer JN . Administrative database concerns: accuracy of International Classification of Diseases, Ninth Revision coding is poor for preoperative anemia in patients undergoing spinal fusion. Spine (Phila Pa 1976) (2014) 39(24):2019–23. doi: 10.1097/BRS.0000000000000598 25202941

[41] Freeman J . Juvenile Rheumatoid Arthritis (JRA): Does JRA Ever Go Away? (2018). Available at: https://www.rheumatoidarthritis.org/ra/juvenile/#:~:text=Juvenile%20chronic%20arthritis%20and%20juvenile%20idiopathic%20arthritis%20are,High%20fevers.%20Rashes%20that%20appear%20with%20fevers.%20Stiffness.

[42] Hunter TM Boytsov NN Zhang X Schroeder K Michaud K Araujo AB . Prevalence of rheumatoid arthritis in the United States adult population in healthcare claims databases, 2004–2014. Rheumatol Int (2017) 37(9):1551–7. doi: 10.1007/s00296-017-3726-1 28455559

[43] Curtis JR Xie F Yun H Bernatsky S Winthrop KL . Real-world comparative risks of herpes virus infections in tofacitinib and biologic-treated patients with rheumatoid arthritis. Ann rheumatic diseases (2016) 75(10):1843–7. doi: 10.1136/annrheumdis-2016-209131 PMC5553444 27113415

[44] Pawar A Desai RJ Solomon DH Ortiz AJS Gale S Bao M . Risk of serious infections in tocilizumab versus other biologic drugs in patients with rheumatoid arthritis: a multidatabase cohort study. Ann rheumatic diseases (2019) 78(4):456–64. doi: 10.1136/annrheumdis-2018-214367 30679153

[45] Kim SC Glynn RJ Giovannucci E Hernández-Díaz S Liu J Feldman S . Risk of high-grade cervical dysplasia and cervical cancer in women with systemic inflammatory diseases: a population-based cohort study. Ann rheumatic diseases (2015) 74(7):1360–7. doi: 10.1136/annrheumdis-2013-204993 PMC4161656 24618265

[46] Stein JD Rahman M Andrews C Ehrlich JR Kamat S Shah M . Evaluation of an algorithm for identifying ocular conditions in electronic health record data. JAMA Ophthalmol (2019) 137(5):491–7. doi: 10.1001/jamaophthalmol.2018.7051 PMC6512255 30789656

[47] Regier DA Kaelber CT Roper MT Rae DS Sartorius N . The ICD-10 clinical field trial for mental and behavioral disorders: results in Canada and the United States. Am J Psychiatry (1994) 151(9):1340–50. doi: 10.1176/ajp.151.9.1340 8067491

