# Supplementary material for: Discrepancies in ICD-9/ICD-10-based codes used to identify three common diseases in cancer patients in real-world settings and their implications for disease classification in breast cancer patients and patients without cancer: a literature review and descriptive study
Source: Front Oncol. 2023 Sep 6;13:1016389. doi: 10.3389/fonc.2023.1016389 (PMC10512179; doi:10.3389/fonc.2023.1016389)
Supplement: Supplementary file 1 [file Table_1.docx]

Supplementary Material

# Supplementary Tables

# **Supplementary Table 1**. Complete Search Strategy for Literature Search in Embase (1980 – 22 February 2021) and Ovid MEDLINE (1946 – 22 February 2021).

| **#** | **Searches** | **Results** |
| --- | --- | --- |
| 1 | *ICD-9/ or *ICD-10/ or *Current Procedural Terminology/ or *Healthcare Common Procedure Coding System/ | 4804 |
| 2 | (code? or coding).ti,ab. | 805070 |
| 3 | 1 and 2 | 2539 |
| 4 | (adverse event? or adverse drug event? or adverse effect? or adverse reaction? or adverse drug reaction?).ti,ab,kw. | 965034 |
| 5 | (validat???? or predict???? or identif????).ti,ab. | 10171688 |
| 6 | 3 and 4 and 5 | 52 |
| 7 | anemia.ab. and anemia.kw,hw. | 157023 |
| 8 | 3 and 7 | 7 |
| 9 | hypertension.ab. and hypertension.kw,hw. | 604702 |
| 10 | 3 and 9 | 21 |
| 11 | arthritis.ab. and arthritis.kw,hw. | 269884 |
| 12 | 3 and 11 | 19 |
| 13 | 6 or 8 or 10 or 12 | 99 |
| 14 | limit 13 to English | 94 |
| 15 | remove duplicates from 14 | 85* |

**Please note that 1 duplicate reference was removed from the final results. The reference count is now 84; ti,title; ab, abstract; kw, key word; hw, subject heading word*

## Supplementary Table 2. Most frequently reported concordant ICD-9 and ICD-10 codes identified in both the literature/web search and 27 CCW Chronic Conditions Algorithm.

| **Disease** | **# References** | **ICD Code(s)** | **Description** |
| --- | --- | --- | --- |
| **ICD-9 Codes** | | | |
| Anemia | 4 | 285.9 | Anemia, unspecified |
|  | 3 | 285.2x | Anemia of chronic illness |
|  | 2 | 280.x  281.x | Iron deficiency anemias  Other deficiency anemias |
| Hypertension | 5 | 401.9 | Malignant essential hypertension |
|  | 4 | 401.0, 401.1, 402.xx, 403.xx, 404.xx, 405.xx | Benign or unspecified essential hypertension  Certain malignant or benign hypertensive heart disease  Hypertensive chronic kidney disease  Hypertensive heart and chronic kidney disease  Secondary hypertension |
| Arthritis | 10 | 714.0 | RA |
|  | 8 | 714.1, 714.2 | Felty’s syndrome  Other RA with visceral or systemic involvement |
|  | 6 | 714.3x | Juvenile chronic polyarthritis |
| **ICD-10 Codes** | | | |
| Anemia | 3 | D50.0 | IDA secondary to blood loss (chronic) |
|  | 2 | D50.8, D50.9, D51.x | Other or unspecified IDA  Certain vitamin B12 deficiency anemias |
| Hypertension | 4 | I11.x | Essential (primary) hypertension  Hypertensive heart disease without HF |
|  | 3 | I12.x, I13.xx, I15.x | Hypertensive CKD  Hypertensive heart and CKD  Secondary hypertension |
| Arthritis | 8 | M05.4xx, M05.5xx, M05.7xx, M05.9 | Rheumatoid myopathy with RA  Rheumatoid polyneuropathy with RA  RA with RF without organ or systems involvement  RA with RF, unspecified |
|  | 7 | M05.0xx, M05.2xx, M05.3xx, M05.6xx, M05.8xx, M06.0xx (excluding M06.0A), M06.8xx (excluding M08.8A), M06.9 | Felty's syndrome  Rheumatoid vasculitis with RA  Rheumatoid heart disease with RA  RA with involvement of other organs and systems  Other RA with RF  RA without RF  Other specified RA, elbow  RA, unspecified |
|  | 6 | M06.2xx, M06.3xx | Rheumatoid bursitis  Rheumatoid nodule |
|  | 5 | M06.1 | Adult-onset Still's disease |
|  | 4 | M16.xx, M17.xx | OA of hip  OA of knee |
|  | 3 | M15.x, M18.xx, M19.xxx (excluding M19.29) | Polyosteoarthritis  OA of first carpometacarpal joint  Other and unspecified OA |

## Supplementary Table 3. Most frequently reported discordant ICD-9 and ICD-10 codes found only in the literature/web search.

| Disease | # References | ICD Code(s) | Description |
| --- | --- | --- | --- |
| ICD 9 Codes | | | |
| Anemia | N/A | | |
| Hypertension | 2 | 642.0x, 642.1x, 642.2x, 642.7x, 642.9x | Certain conditions complicating pregnancy, childbirth, or the puerperium (including benign essential hypertension, hypertension secondary to renal disease, other or unspecified pre-existing hypertension)  Pre-eclampsia or eclampsia superimposed on pre-existing hypertension |
| Arthritis | 7 | 714.4, 714.8x | Chronic post-rheumatic arthropathy  Other specified inflammatory polyarthropathies (including rheumatoid lung) |
|  | 6 | 714.9 | Unspecified inflammatory polyarthropathy |
| ICD-10 Codes | | | |
| Anemia | N/A | | |
| Hypertension | N/A | | |
| Arthritis | 7 | M05.10, M05.11x, M05.13x, M05.14x, M05.15x, M05.61, M05.171, M05.172, | Certain codes for rheumatoid lung disease with RA  Other RA with RF of other unspecified site  RA without RF of other unspecified site |
|  | 5 | M06.4 | Inflammatory polyarthropathy |

OA, osteoarthritis; RA, rheumatoid arthritis; RF, rheumatoid factor
